# Dysregulated Monocyte and Neutrophil Functional Phenotype in Infants With Neonatal Encephalopathy Requiring Therapeutic Hypothermia

**DOI:** 10.3389/fped.2020.598724

**Published:** 2021-02-15

**Authors:** Mary Isabel O'Dea, Lynne Kelly, Ellen McKenna, Ashanty M. Melo, Megan Ni Bhroin, Tim Hurley, Angela T. Byrne, Gabrielle Colleran, Claudine Vavasseur, Afif El-Khuffash, Jan Miletin, John Murphy, Fionnuala Hickey, Eleanor J. Molloy

**Affiliations:** ^1^Department of Paediatrics and Neonatology, Coombe Women & Infants University Hospital, Dublin, Ireland; ^2^Trinity Translational Medicine Institute, Trinity College Dublin, Dublin, Ireland; ^3^National Children's Research Centre (NCRC), Crumlin, Ireland; ^4^Trinity College Institute of Neuroscience and Cognitive Systems Group, Discipline of Psychiatry, School of Medicine, Trinity College Dublin, Dublin, Ireland; ^5^Department of Paediatrics and Child Health, Trinity College Dublin, Dublin, Ireland; ^6^Children's Health Ireland at Crumlin, Dublin, Ireland; ^7^Department of Radiology, National Maternity Hospital, Dublin, Ireland; ^8^National Maternity Hospital, Dublin, Ireland; ^9^Rotunda Hospital, Dublin, Ireland; ^10^Trinity Health Kidney Centre, Faculty of Health Sciences, School of Medicine, Trinity College Dublin, Dublin, Ireland; ^11^Our Lady's Children's Hospital (CHI), Crumlin, Ireland; ^12^Department of Paediatrics, Tallaght University Hospital, Dublin, Ireland

**Keywords:** neuroimmunology, monocytes, neutrophils, neonatal encephalopathy, innate immunity

## Abstract

Neonatal encephalopathy (NE) is a significant cause of morbidity and mortality. Persistent inflammation and activation of leukocytes mediate brain injury in NE. The standard of care for NE, therapeutic hypothermia (TH), does not improve outcomes in nearly half of moderate to severe cases, resulting in the need for new adjuvant therapies, and immunomodulation holds promise. Our objective was to explore systemic leukocyte phenotype in infants with NE and healthy controls in response to lipopolysaccharide (LPS). Twenty-four infants with NE (NE II-20; NE III = 4) requiring TH and 17 term neonatal controls were enrolled, and blood samples were analyzed between days 1 and 4 of life at a mean (SD) timepoint of 2.1 (± 0.81) days of postnatal life at the time of the routine phlebotomy. Leukocyte cell surface expression levels of Toll-like receptor 4, NADPH oxidase (NOX2), CD11b, mitochondrial mass, and mitochondrial superoxide production were measured by flow cytometry. Gene expression of TRIF (TIR domain–containing adapter-inducing interferon-β), MyD88 and IRAK4 was measured by reverse transcription–polymerase chain reaction. Infants with NE had significantly lower expression of neutrophil CD11b and NOX2 with LPS stimulation compared to healthy term controls. Mitochondrial mass in neutrophils and monocytes was significantly increased in NE infants with LPS compared to controls, potentially indicating a dysregulated metabolism. Infants with NE had significantly lower IRAK4 at baseline than controls. NE infants display a dysregulated inflammatory response compared to healthy infants, with LPS hyporesponsiveness to CD11b and NOX2 and decreased IRAK4 gene expression. This dysregulated immune profile may indicate an adaptable response to limit hyperinflammation.

## Introduction

The neonatal period is the most vulnerable time for childhood mortality, 44% of deaths in the population younger than 5 years occur during this time, with 29% of these attributed to neonatal encephalopathy (NE) (1). The estimated global burden of NE is 1.15 million, with 96% of NE infants born in low- and middle-income countries (2). There has been significant progress in the management of NE with the development of therapeutic hypothermia (TH) as the standard of care; however, morbidity and mortality remain high (3).

Immune dysfunction is well-described in NE; nevertheless, prior to developing new immunomodulatory therapies, further understanding of the inflammatory phenotype is crucial (4, 5). For instance, altered levels of proinflammatory and anti-inflammatory cytokines and a dysregulated cellular inflammatory response to endotoxin have been demonstrated in NE (1, 2, 6). Different immunomodulators have been postulated to target inflammation, excitotoxicity, and oxidative stress in NE, such as the nucleotide-binding domain-like receptor protein 3 (NLRP3) inflammasome (7).

Neutrophils and monocytes are involved in systemic inflammation in NE (1). Toll-like receptor 4 (TLR-4) is an immune cell surface receptor involved in recognizing endotoxin, and CD11b is a marker of cell activation. Myeloid differentiation primary response (MyD88) and TIR domain–containing adapter-inducing interferon-β (TRIF) are adaptors that bind to the intracellular domains of the TLR and interleukin-1 receptor families, linking them to IL-1R–associated kinase (IRAK) (8). Activation of IRAK activates downstream inflammatory pathways leading to expression of proinflammatory cytokines: tumor necrosis factor α, interleukin 1β (IL-1β), IL-6, and interferon γ (8).

Mitochondria are a source of reactive oxygen species (ROS) in the cell; in many pathologies, ROS contributes to mitochondrial damage (9). Glycolysis and oxidative phosphorylation support monocyte function; during inflammation, the mitochondria allows macrophages to switch from glycolysis to oxidative phosphorylation (9). Neutrophil mitochondria depend on anaerobic glycolysis and are adapted to working under hypoxia (10).

We hypothesized that infants with NE may have an altered inflammatory phenotype in terms of markers of activation CD11b, TLR-4 [lipopolysaccharide (LPS) responsiveness], and NOX2. We examined underlying mechanisms of the immunophenotype in NE at the mRNA level for TLR-4 signaling and examined the mechanistic role of the mitochondria in neutrophils and monocytes by examining superoxide production and mitochondrial mass.

## Methods

### Study Population

This study was approved by the ethics committees of the three Dublin Maternity Hospitals, which are all tertiary neonatal intensive care units (NICUs) and national referral centers for TH. Families received verbal and documented information on the study, and written consent was obtained prior to recruitment.

We have previously recruited several distinct cohorts of infants with NE and controls with criteria as described (2, 11). The NE severity was classified by Sarnat staging (12). Infants with NE had a magnetic resonance imaging (MRI) of the brain completed and scored as per Barkovich classification, a scoring system for the assessment of brain injury that is predictive of motor outcome. The score evaluates basal ganglia injury, watershed injury, combined injury score, and summation score (13). Infants with congenital abnormalities or evidence of maternal substance abuse were excluded. Asymptomatic well-term infants undergoing routine phlebotomy were included as controls, but infants undergoing sepsis evaluations or receiving phototherapy for jaundice were excluded.

### Blood Sampling

Whole-blood sampling was performed following informed parental consent. Sampling was performed using aseptic technique via central and peripheral arterial lines and via venous sampling at times of routine patient phlebotomy at one timepoint during their NICU course. The blood was collected in sodium citrate tubes and processed immediately. The volume taken was 1–1.4 mL, which was approved by the ethics committee, as a volume that would not affect the infant's hemodynamic status.

### Cell Surface Antigen Expression

The expression of TLR4, NOX2, and CD11b on the surface of neutrophils and monocytes was evaluated by flow cytometry. Whole blood (200 μL) was treated in 100-μL aliquots incubated at 37°C for 1 h untreated (vehicle) or with 10 ng/mL LPS (SIGMA Life Science, Ireland). Fluorochrome-conjugated monoclonal antibodies (mAb) specific for humans CD14-PerCP, CD15-PECy7, NOX2-FITC, CD66b-Pacific Blue, TLR4-APC (BioLegend®, USA), and CD11b-PE (BD Biosciences, UK) were used. The whole blood was then stained with mAb for 15 min. Red blood cells were lysed with BD lysis buffer. Cells were acquired on a FACSCanto II flow cytometer (BD Bioscience) and analyzed using FlowJo version 10 (Tree Star) (2, 14). Neutrophils were delineated based on SSC-A and CD66b^+^ and monocytes based on SSC-A, CD66b^−^, and CD14^+^ (15, 16). Monocytes were subdivided into classical, intermediate, and non-classical subtypes. Monocyte subsets were identified as classical: CD14^high^CD16^neg/low^; intermediate: CD14^high^CD16^high^; non-classical: CD14^low^CD16^high^. A minimum of 10,000 events were collected, and relative expression of antigens was expressed as mean fluorescence intensity (MFI).

### Mitochondrial Mass and ROS

MitoTracker green (MTG) was used to assess mitochondrial mass of leukocytes. MitoSox red was used to assess mitochondrial superoxide production of leukocytes. Generation of mitochondrial ROS (mtROS) and mitochondrial mass were evaluated using flow cytometry. Whole blood was incubated at 37°C for 1 h untreated (vehicle) or with 10 ng/mL LPS in the presence of MTG dye (1.4 μM) and MitoSox red reagent (15 μM) (ThermoFisher Scientific). Samples were stained with mAb for CD15- PECy7, CD66b-PB (BioLegend®, USA), CD14-APC (Beckman Coulter), and CD16^−^V500 (BD Biosciences, UK) for 15 min. BD lysis buffer was used to lyse red blood cells. Cells were acquired on a BD FACSCanto II flow cytometer. Fluorescence minus one (FMO) controls were used to set gates. Mitochondrial mass and superoxide were quantified based on MFI of MTG and MitoSOX red, respectively (17). Data were analyzed using FlowJo software version 10.

### Reverse Transcription–Polymerase Chain Reaction

Gene expression of TRIF, IRAK4, and MyD88 in whole blood in the presence or absence of LPS was examined using reverse transcription–polymerase chain reaction (RT-PCR). Following whole-blood incubation of samples with LPS, RNA extraction was performed using Ribopure blood kit as per manufacturer (ThermoFisher). RNA purity and concentration were measured using a NanoDrop ND-100 spectrophotometer and analyzed using ND-1000 version 3.1.2 software. Total RNA, 1 μg, was reverse transcribed to single-stranded cDNA using the High-Capacity cDNA Archive Kit (Applied Biosystems) as per manufacturer. The settings for amplification were 10 min at 25°C, 120 min at 37°C, and 5 s at 85°C and holding at 4°C. TaqMan® primer and probe combinations were used to detect gene expression of MyD88 (NM_001172567.1), TRIF (NM_182919.3), and IRAK4 (NM_001114182.2). Thermal cycling conditions were as follows: 2 min at 50°C, 10 min at 95°C, and, for 40 cycles, 24 s at 95°C and 1 min at 60°C, using a 7900HT Fast Real-Time PCR System. Relative quantification values were calculated using the 2^−ΔΔCt^ method (16).

### Statistics

Statistical analysis was performed on GraphPad PRISM version 8 using unpaired (unmatched) or paired (matched), two-tailed *t*-tests to compare mean results between two independent cohorts. The Kolmogorov–Smirnov test was used to check normality. Significance was defined as *p* < 0.05. Results shown are expressed as mean ± standard error of the mean.

## Results

### Patient Demographics

Twenty-four infants with NE (NE II, *n* = 20; NE III, *n* = 4) who required TH and 17 term neonatal controls were enrolled. The infants with NE had their phlebotomy performed at a mean (SD) timepoint of 2.1 (± 0.81) days of postnatal life, on day 1 (*n* = 5), day 2 (*n* = 14), day 3 (*n* = 3), and day 4 (*n* = 2).

In the NE group, the mean (SD) Apgar scores were 2.9 (2.0) and 5.0 (2.3) at 1 and 5 min, respectively, and no infants died. The mean (SD) birth weight was 3.4 (0.5) kg, and there were 16 male infants. Infants with NE had an MRI brain, assessed with the Barkovich scoring classification as follows: eight infants had a normal scan, eight infants had isolated watershed injury, and no infants had an isolated basal ganglia injury. Seven infants had both watershed and basal ganglia injury. Two infants did not have an MRI Barkovich score completed.

The term control infants had a mean (SD) gestation of 39.2 (1.6) weeks, birth weight of 3.3 (0.5) kg, and Apgar scores of 9 and 10 (± 0) at 5 and 10 min, and the group included five male infants.

### Leukocyte Cell Surface Markers

**Figure 1:**
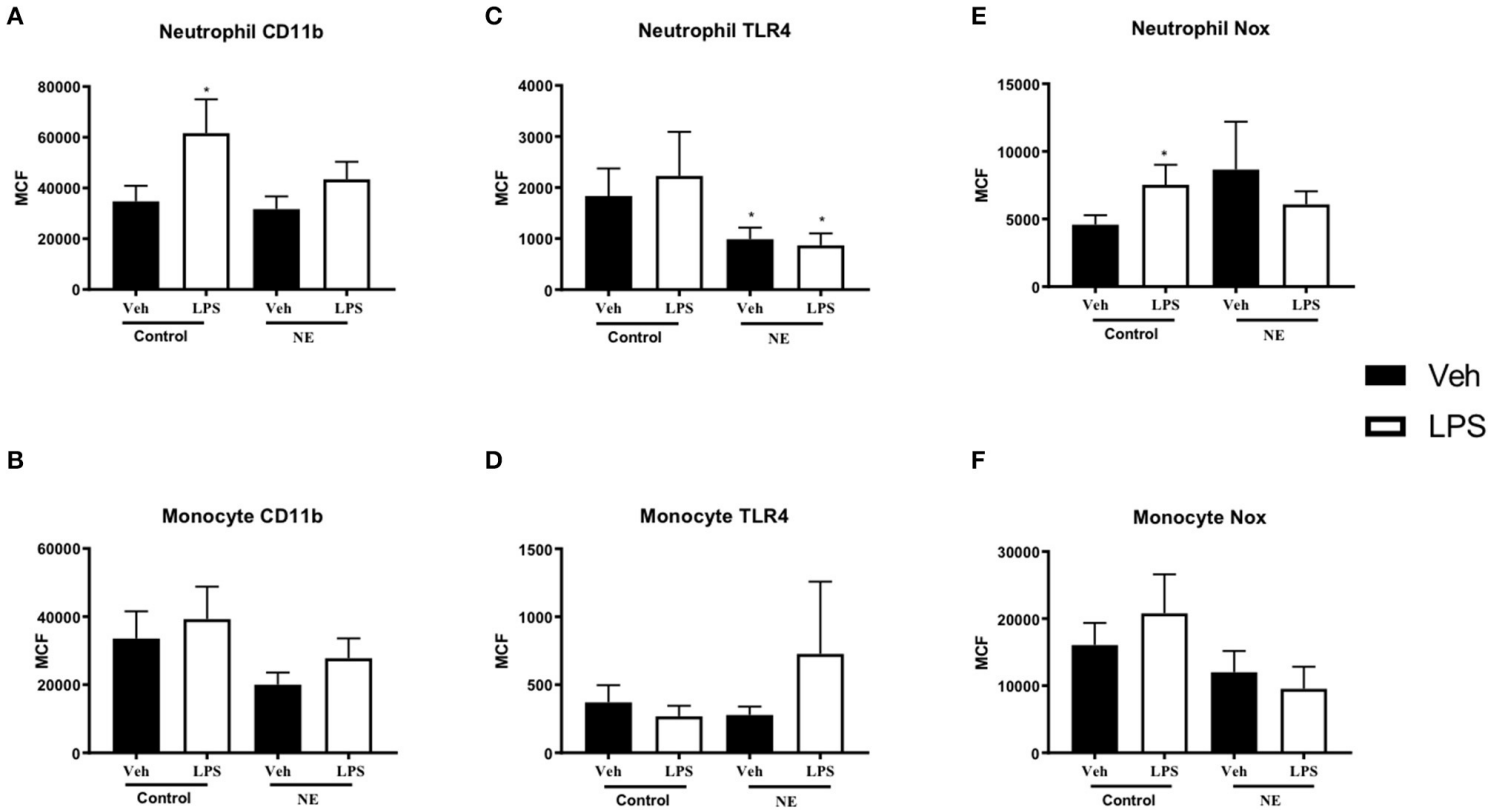
CD11b, TLR-4, and NOX2 expression in healthy controls and in neonatal encephalopathy. Whole blood from NE patients and controls was stimulated with LPS and leukocyte markers were analyzed by flow cytometry. Graph shows MCF of CD11b, TLR-4, and NOX2 on neutrophils and monocytes. Neutrophils and monocytes were identified based on their size and granularity (FSC, forward scatter; and SSC, side scatter, respectively), followed by labeling with CD66b^+^ for granulocytes, and CD66b^−^ and CD14^+^ for monocytes. Results are expressed as mean channel fluorescence (MCF); “veh” refers to unstimulated (*p < 0.05 using unpaired, two-tailed t-test). A significant increase in neutrophil CD11b expression following LPS stimulation was observed in controls (*p* = 0.021) but not in NE infants **(A)**. Neutrophil TLR4 was lower in infants with NE at baseline compared to controls and following LPS stimulation (*p* = 0.05) **(C)**. Significantly elevated neutrophil NOX2 expression with LPS was demonstrated in controls but not in NE infants (*p* = 0.046) **(E)**. Monocyte Cd11b, TLR4, and Nox expression did not differ between controls and in NE at baseline nor with LPS stimulation **(B,D,F)**.

A significant increase in neutrophil CD11b expression following LPS stimulation was observed in controls (*p* = 0.021) but not in NE infants (Figure 1A). No difference in monocyte CD11b expression between healthy controls and infants with NE was observed (Figure 1B). Neutrophil TLR4 was lower in infants with NE at baseline compared to controls and following LPS stimulation (*p* = 0.05) (Figure 1C). Significantly elevated neutrophil NOX2 expression with LPS was demonstrated in controls but not in NE infants (*p* = 0.046) (Figure 1E).

Infants with NE showed relative LPS hyporesponsiveness to neutrophil CD11b and NOX2 expression indicating an altered inflammatory response.

### Mitochondrial Mass and mtROS

**Figure 2:**
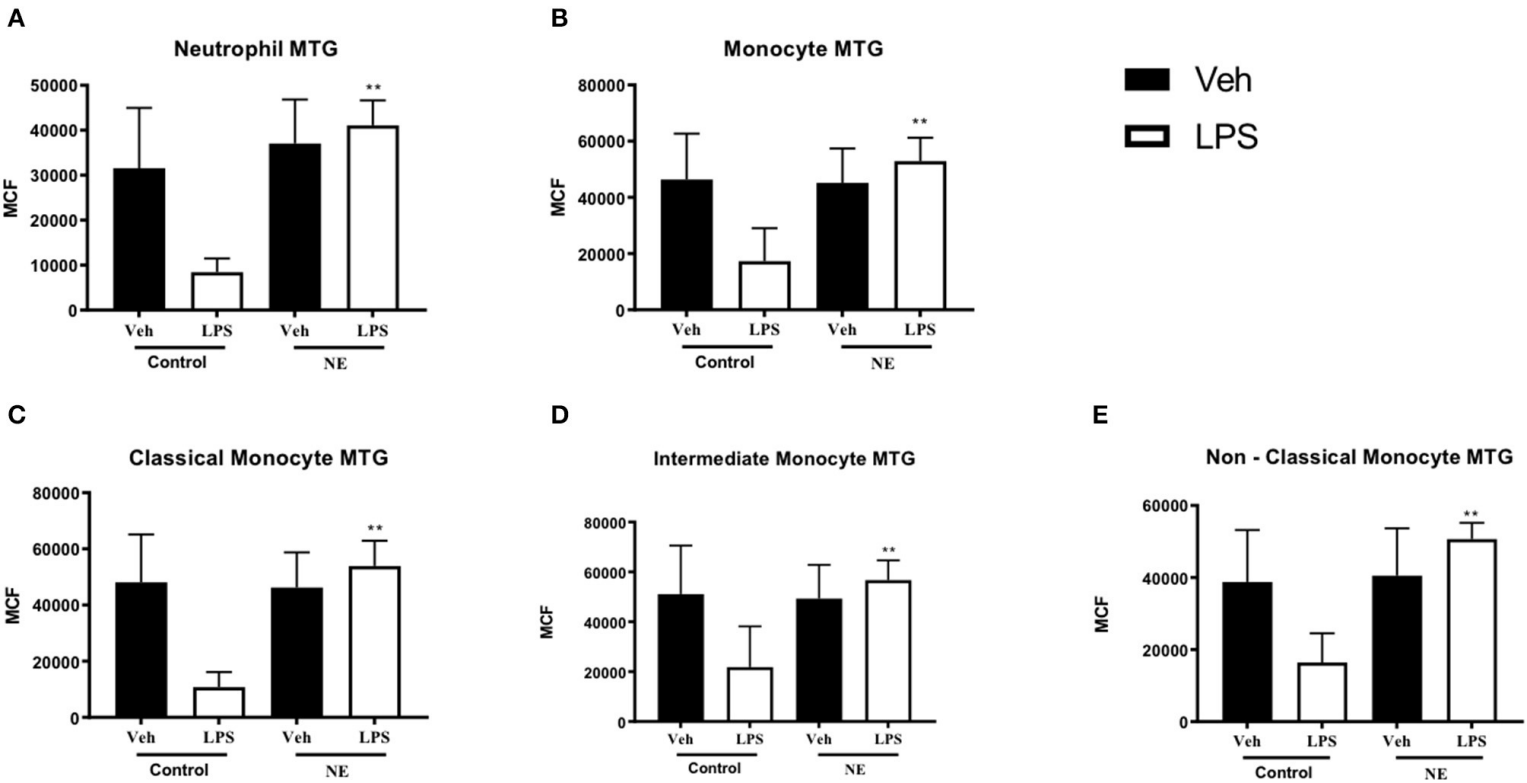
Neutrophil and monocyte mitochondrial mass in healthy term controls compared to NE. MitoTracker (MTG) green was used to measure mitochondrial mass of leukocytes in whole blood from NE patients and controls following LPS stimulation. Graphs show MTG of **(A)** neutrophils, **(B)** monocytes, **(C)** classical monocyte MTG, **(D)** intermediate monocyte MTG, **(E)** non-classical monocyte MTG, in healthy term controls (*n* = 4–5) and NE infants (*n* = 4–5). CD14 and CD16 FMO ensured a standardized way of dividing monocytes into distinct populations in the context of the data spread due to the multiple fluorochromes in the panel. Monocytes were divided into classical, non-classical, and intermediate monocytes. Results are expressed as mean channel fluorescence (MCF); “veh” refers to unstimulated (*p < 0.05, **p < 0.01 using unpaired two-tailed *t*-test).

Following treatment with LPS, neutrophils (*p* = 0.002, Figure 2A) and monocytes (*p* = 0.038. Figure 2B) from infants with NE had significantly higher mitochondrial mass in comparison with healthy term controls. Following treatment with LPS, all monocyte subsets (Figures 2C–E) from infants with NE had significantly higher mitochondrial mass in comparison with controls (all *p* < 0.05).

Infants with NE displayed higher mitochondrial mass than controls in response to LPS, which may potentially demonstrate that infants with NE have higher levels of metabolism and superoxide production than healthy controls.

**Figure 3:**
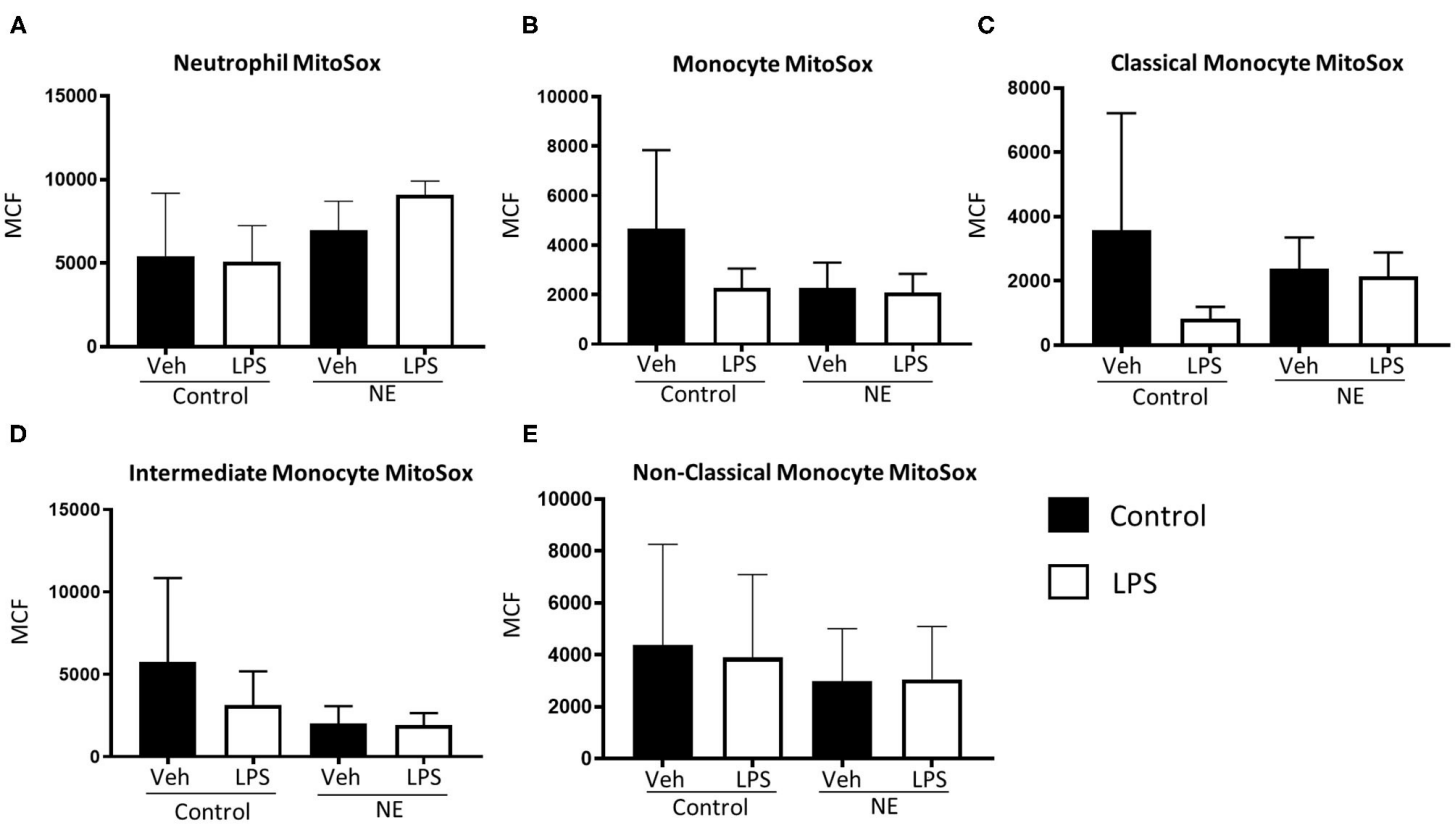
Mitochondrial superoxide in neutrophils and monocytes in controls vs. NE. MitoSOX red was used to assess mitochondrial superoxide production of leukocytes in whole blood from NE patients and controls following LPS stimulation. Graphs show MitoSOX of **(A)** neutrophils, **(B)** monocytes, **(C)** classical monocyte MTG, **(D)** intermediate monocyte MTG, **(E)** non-classical monocyte MTG in healthy term controls (*n* = 3–5) and NE infants (*n* = 4–5). Results are expressed as mean channel fluorescence (MCF); “veh” refers to unstimulated.

There were no significant differences in mtROS production for neutrophils or monocytes between controls and infants with NE (Figure 3).

### TRIF, IRAK4, and MyD88

**Figure 4:**
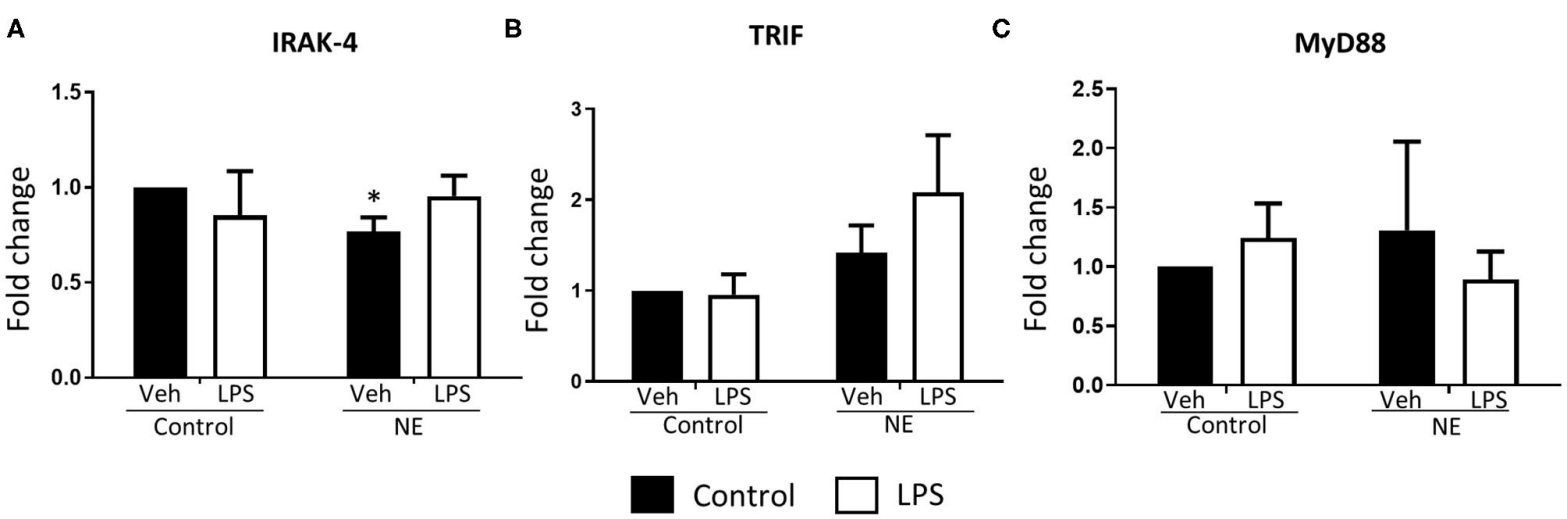
Gene expression of TLR signaling molecules in term controls and neonatal encephalopathy. Gene expression of IRAK4, TRIF, and MyD88 in whole blood in the presence or absence of LPS was measured using RT-PCR. Graphs showing **(A)** interleukin-1 receptor-associated kinase 4 (IRAK4), **(B)** TIR domain–containing adapter-inducing interferon-β (TRIF), and **(C)** myeloid differentiation primary response 88 (MyD88) in term control (n = 5) and neonatal encephalopathy (*n* = 5). The results are displayed as fold change expression when the control vehicle is normalized to one. They are represented as fold change at baseline and in response to endotoxin stimulation. The error bars represent standard error of the mean; “veh” refers to unstimulated (*p < 0.05 using unpaired two-tailed *t*-test).

There was significantly lower IRAK4 expression in NE infants at baseline than control infants (*p* = 0.014) with no differences detected upon LPS stimulation (Figure 4A). No difference in TRIF or MyD88 expression between infants with NE and controls was observed (Figures 4B,C). Lower IRAK4 gene expression in NE infants compared to control infants suggests a possible IRAK4 deficiency in NE infants.

## Discussion

We found that infants with NE undergoing TH displayed neutrophil and monocyte LPS hyporesponsiveness for surface expression of NOX2 and CD11b. IRAK4 gene expression was lower in NE at baseline, whereas mitochondrial mass was higher in NE with LPS compared to controls.

CD11b was significantly increased on the surface of neutrophils in healthy controls but not in infants with NE. Neutrophils from infants with NE II/III had decreased CD11b in response to LPS compared to controls (18). Neutrophils are the earliest leukocyte to initiate the inflammatory response; hypoxia can delay neutrophil apoptosis, causing a persistent inflammatory response, increased ROS production, and CD11b activation (18). O'Hare et al. demonstrated higher monocyte and neutrophil CD11b in NE infants compared to adult blood at baseline and in response to LPS stimulation but lacked neonatal controls; hence, the comparator group was not as clinically relevant as our own study with neonatal controls (1). The different comparator group makes it difficult to draw significant comparisons between the studies. Children in pediatric intensive care had decreased leukocyte CD11b LPS responses compared to healthy adult and pediatric controls, correlating with our findings (19).

NOX2 expression was significantly elevated with LPS in control infants' neutrophils but not in NE neutrophils (Figure 1). NADPH oxidase increases upon hypoxia–ischemia and contributes to brain injury by production of ROS (10). Following LPS stimulation *ex vivo*, neonates with severe brain injury produced significantly higher systemic neutrophil ROS compared to mildly affected neonates (NE 0/I) at 72–96 h and day 7 of life, but the study lacked neonatal controls (20). Our data may suggest a potential neuroprotective role of NOX2 in healthy infants, and a lack of increase in NE neutrophil NOX2 may suggest these infants cannot mount a protective response.

Neutrophil TLR-4 surface expression was lower in NE than control at baseline and after LPS stimulation. TLR-4 is upregulated with endotoxin in brain injury *in vivo* (14). TLR-4 knockout mice have lower infarct size in stroke model compared to wild type, suggesting that antagonizing TLR-4 may have a role in neuroprotection (21).

mtROS production via neutrophils and monocytes was similar in infants with NE and controls at baseline and with LPS stimulation. ROS can cause significant tissue damage and may be mediated by NADPH oxidase, mitochondrial dysfunction, and hypoxia-inducible factor 1α upregulation. Mitochondria are vulnerable in infants with NE as their capacity to scavenge is overcome by high levels of ROS, initiating mitochondrial permeability transition and oxidative stress (22). Neutrophil mtROS production increased in pediatric patients with systemic lupus erythematosus compared to healthy pediatric controls (23). Mitochondrial autophagy, the ability of mitochondria to maintain homeostasis and adapt to stress, may explain the relatively few differences in NE and controls (24). However, the predominant form of ROS in neutrophils is produced by NADPH oxidase; not mitochondria (20) but mtROS are involved in NOX2 activation. Exocytosis of both primary and secondary granules, and delayed apoptosis and contributes to excessive activation of neutrophils (25). We have previously shown that neonates with abnormal neuroimaging and/or severe NE had increased neutrophil total ROS as well as CD11b and TLR-4 expression (1). An augmented neutrophil total ROS response in infants with NE post-LPS compared to neonatal controls was reduced significantly by activated protein C (26). In addition, although there is similar neutrophil ROS production in preterm and term neonates with high levels of total ROS in the neonatal controls (27). Neutrophils and monocytes of NE infants showed a significantly increased mitochondrial mass than controls with LPS. Endotoxin sensitizes the immature brain to damage in animal models, and infants exposed to *in utero* inflammation by infection have a higher risk of NE (28). Metabolic dysfunction is a hallmark of NE. In the immature brain, mitochondrial swelling results from excessive calcium influx leading to increased superoxide production (28). Elevated mitochondrial mass of leukocytes may be indicative of brain injury typical of NE.

IRAK4 expression was significantly lower in NE than in controls at baseline but did not change with LPS stimulation. There were no differences in TRIF and MyD88 between NE and controls. MyD88, TRIF, and IRAK are adaptor molecules that activate downstream inflammatory responses following TLR activation (8). IRAK inhibition is protective in a necrotizing enterocolitis animal model by preventing the release of proinflammatory cytokines and reducing intestinal damage (29). IRAK4 deficiency in humans impairs TLR innate immunity and causes increased susceptibility to bacterial infections including *Staphylococcus aureus* and *Streptococcus pneumoniae* (30). In most patients with IRAK4 deficiency, the first infection presents before 2 years of age (30). Our results suggest that NE infants are more likely to have IRAK4 deficiency than controls and may have increased susceptibility to infection. An IRAK4 deficiency may indicate an inability of NE infants to mount a proper immune response. Whether this reduction in IRAK4 is a contributing factor or consequence of NE remains to be determined in future work.

Infants with NE were LPS-hyporesponsive to NOX2 and CD11b expression and displayed significantly lower expression of IRAK4 compared to neonatal controls. Neutrophils, monocytes, classical monocytes, and non-classical monocytes showed significantly higher mitochondrial mass in NE than controls with LPS. School-age children with a history of NE have significantly altered cytokine responses post-NE compared to controls (31). This dysregulation in immune function may increase susceptibility to infection and correlate with persistent inflammation in childhood (32). Targeting immune function may protect infants with NE from morbidity. In a piglet model, stimulation with LPS 4 h before hypoxia leads to increased mortality and brain cell death (32).

## Conclusions

NE infants undergoing TH have an altered inflammatory response compared to healthy infants, with LPS hyporesponsiveness to CD11b and NOX2, which may indicate a reduced ability to mount an immune response to infection. NE infants showed a higher mitochondrial mass, which may demonstrate higher metabolic dysfunction and a higher production of superoxide, which may be associated with brain injury in NE. Lower IRAK4 gene expression in the NE infants implies that NE infants may have an increased susceptibility to infection, demonstrating that NE infants have an adaptable response to limit hyperinflammation. This article is novel in the use of neonatal blood samples instead of cord blood as a comparator group.

The results are limited by the variability in the timing of phlebotomy, with a median of 2.1 (± 0.81) and two infants having sampling done post-TH. Future directions include comparison of the infant's immunophenotype during and after TH. Future studies are needed to reveal any therapeutic potential of the identified targets, such as CD11b, NOX, and IRAK4.

## Data Availability Statement

The raw data supporting the conclusions of this article will be made available by the authors, without undue reservation.

## Ethics Statement

The studies involving human participants were reviewed and approved by Dublin Maternity Hospitals. Written informed consent to participate in this study was provided by the participants' legal guardian/next of kin.

## Author Contributions

MO'D performed the acquisition, analysis and interpretation of data, carried out statistical analysis, and wrote the manuscript. EJM conceived the project, analysis and co-wrote the manuscript, and supervised the experiments. LK, EM, AM, CV, AE-K, JMi, JMu, and FH assisted in data acquisition, writing, and revision of the manuscript. TH, MN, AB, and GC scored each MRI brain as per Barkovich scoring and that result is now included in the paper post reviewers request. All authors contributed to the article and approved the submitted version.

## Conflict of Interest

The authors declare that the research was conducted in the absence of any commercial or financial relationships that could be construed as a potential conflict of interest.

## Supplementary Material

Supplementary Figure 1 Gating strategy for isolation of granulocytes and monocyte subpopulations. Neutrophils were delineated based on SSC-A and CD66b + positivity. Monocytes were defined based on SSC-A, CD66b^−^, and subsets based on relative CD14^+^CD16^+^ populations; classical (CD14^+^/CD16^−^), intermediate (CD14^+^/CD16^+^), non-classical (CD14dim/CD16^+^).

## Abbreviations

IL: Interleukin
IRAK: IL-1R–associated kinase
LPS: Lipopolysaccharide
MFI: Mean fluorescence intensity
MTG: MitoTracker green
MyD88: Myeloid differentiation primary response
NADPH: Nicotinamide adenine dinucleotide phosphate
NE: Neonatal encephalopathy
NLRP3: Nucleotide-binding domain-like receptor protein 3
NOX2: NADPH oxidase
ROS: Reactive oxygen species
TH: Therapeutic hypothermia
TLR-4: Toll-like receptor 4
TRIF: TIR domain–containing adapter-inducing interferon-β.
