# Supplementary figures and images for: Dysregulated Monocyte and Neutrophil Functional Phenotype in Infants With Neonatal Encephalopathy Requiring Therapeutic Hypothermia
Source: Front Pediatr. 2021 Feb 15;8:598724. doi: 10.3389/fped.2020.598724 (PMC7917189; doi:10.3389/fped.2020.598724)

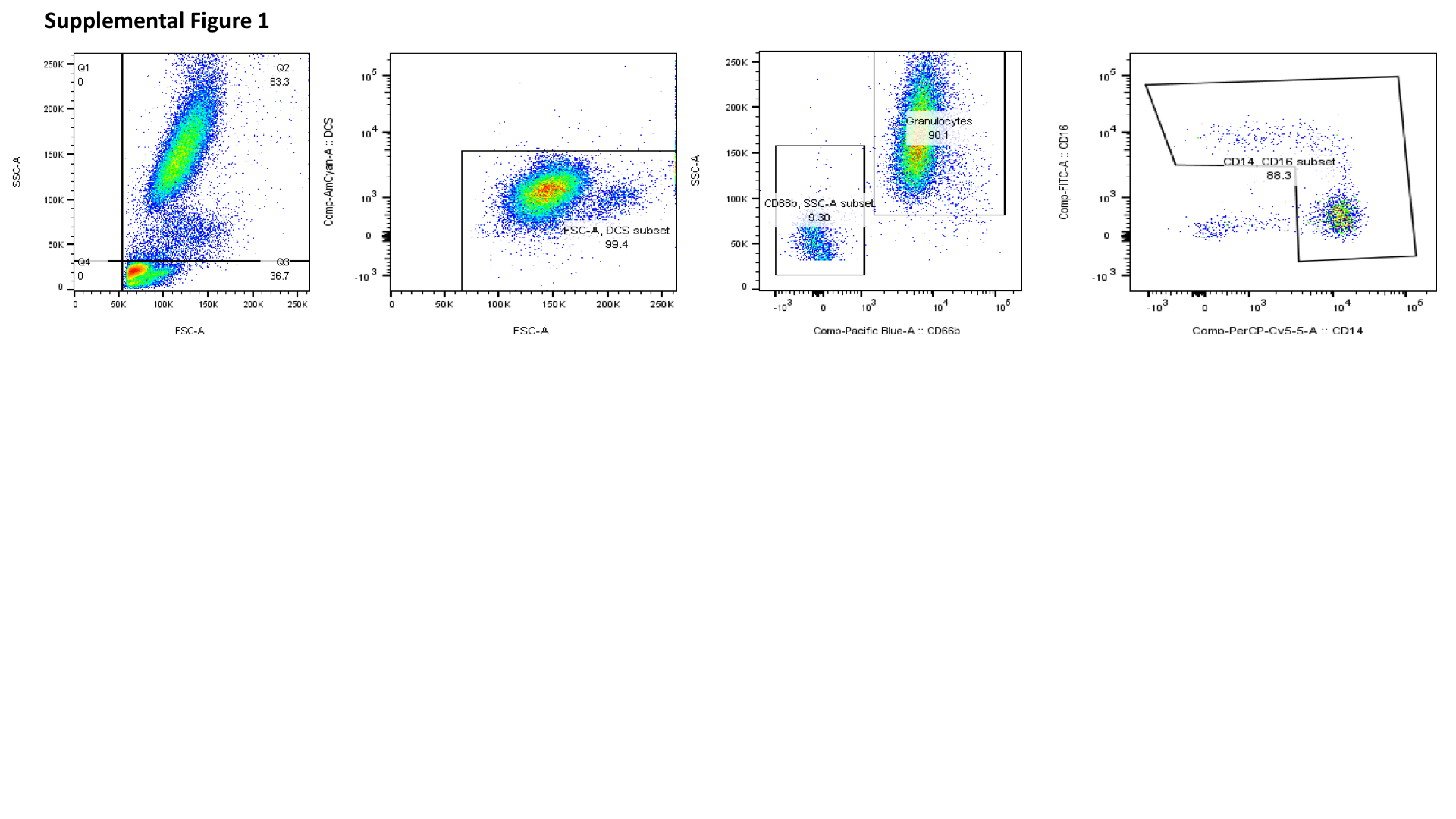

Supplement: Supplementary Figure 1 — Gating strategy for isolation of granulocytes and monocyte subpopulations. Neutrophils were delineated based on SSC-A and CD66b + positivity. Monocytes were defined based on SSC-A, CD66b−, and subsets based on relative CD14+CD16+ populations; classical (CD14+/CD16−), intermediate (CD14+/CD16+), non-classical (CD14dim/CD16+). [file Image_1.tiff]
